# Metastability in the mixing/demixing of two species with reciprocally concentration-dependent diffusivity

**DOI:** 10.3389/fnetp.2025.1612495

**Published:** 2025-11-17

**Authors:** Alexander B. Neiman, Xiaochen Dong, Benjamin Lindner

**Affiliations:** 1 Department of Physics and Astronomy, Ohio University, Athens, OH, United States; 2 Neuroscience Program, Ohio University, Athens, OH, United States; 3 Bernstein Center for Computational Neuroscience Berlin, Berlin, Germany; 4 Department of Physics, Humboldt University Berlin, Berlin, Germany

**Keywords:** Brownian particles, pattern formation, bistability, active matter, noise-induced switching, network physiology, self-organization

## Abstract

It has been shown before that two species of diffusing particles can separate from each other by the mechanism of reciprocally concentration-dependent diffusivity: the presence of one species amplifies the diffusion coefficient of the respective other one, causing the two densities of particles to separate spontaneously. In a minimal model, this could be observed with a quadratic dependence of the diffusion coefficient on the density of the other species. Here, we consider a more realistic sigmoidal dependence as a logistic function on the other particle’s density averaged over a finite sensing radius. The sigmoidal dependence accounts for the saturation effects of the diffusion coefficients, which cannot grow without bounds. We show that sigmoidal (logistic) cross-diffusion leads to a new regime in which a homogeneous disordered (well-mixed) state and a spontaneously separated ordered (demixed) state coexist, forming two long-lived metastable configurations. In systems with a finite number of particles, random fluctuations induce repeated transitions between these two states. By tracking an order parameter that distinguishes mixed from demixed phases, we measure the corresponding mean residence in each state and demonstrate that one lifetime increases and the other decreases as the logistic coupling parameter is varied. The system thus displays typical features of a first-order phase transition, including hysteresis for large particle numbers. In addition, we compute the correlation time of the order parameter and show that it exhibits a pronounced maximum within the bistable parameter range, growing exponentially with the total particle number.

## 1 Introduction

The emergence of spatiotemporal structures in suspensions of active particles is an important topic in the current research in nonequilibrium statistical physics (Romanczuk et al., 2012; Marchetti et al., 2013; Costanzo et al., 2014; Grossmann et al., 2014; O’Keeffe et al., 2017; Granek et al., 2024). Interestingly, in such settings, order in terms of separation of different sorts of particles may emerge other than by repelling or attracting forces but via the mutual control of motility.

A particularly striking example is the demixing of two different strains of *Escherichia coli* bacteria with reciprocal motility interaction, studied experimentally and in a reaction-diffusion model by Curatolo et al. (2020). A central part of the mechanism of particle separation was a mutual amplification of the diffusion strength caused by the presence of the respective other bacteria. This could be mimicked in the paper by Schimansky-Geier et al. (2021), which presented, simulated, and studied analytically a minimal model in which two populations of overdamped Brownian particles mutually control their strength of diffusivity (the noise intensity of driving fluctuations increases with the concentration of the respective other sort of particles). The dependence of the diffusion coefficient has to be strong enough (qualitatively and quantitatively) to observe a demixing of the two population densities.

Here, we study a variant of the model suggested by Schimansky-Geier et al. (2021) and demonstrate a novel bistability between a well-mixed and a demixed state that results in a system with finite particles in stochastic transitions between an ordered and a disordered state. This result might be interesting and also observable experimentally when the reciprocal motility interaction is particularly tailored.

Our paper is organized as follows. In the next section, we introduce the studied model, focusing on the novel aspect, the sigmoidal diffusion coefficient. In Section 3, we derive the conditions under which a single demixed state, a single homogeneous (well-mixed) state, and bistability between both states can be observed in the model and present the system bifurcation diagram. In Section 4, we show and discuss the results of simulations in the bistable parameter regime, in which we measure the mean times that the system spends in the two bistable states and inspect how these times depend on the bifurcation parameter. We conclude in Section 5 with a discussion of our results.

## 2 Model and measures

We consider two populations of Brownian particles, A and B, each of size N , that diffuse on an interval [−1,1] with reflecting boundary conditions at x=±1 according to

$$\begin{array}{cc} \frac{dx_{A,i}}{dt} & =\sqrt{f\left({\tilde{p}}_{B}\left(x_{A,i},t\right)\right)}\;\xi_{A,i}\left(t\right),\\ \frac{dx_{B,j}}{dt} & =\sqrt{f\left({\tilde{p}}_{A}\left(x_{B,j},t\right)\right)}\;\xi_{B,j}\left(t\right). \end{array}$$ (1)

Here i,j=1,…,N , $\xi_{A,i}\left(t\right)$ and $\xi_{B,j}\left(t\right)$ are white Gaussian noise sources with $\langle \xi_{F,i}\left(t\right)\xi_{G,i}\left(t^{\prime }\right)\rangle =\delta_{F,G}\delta_{i,j}\delta \left(t-t^{\prime }\right)\;with\;F,G\in A,B$ , and the equations are interpreted in the Ito sense (Gardiner, 1985). The intensity by which these fluctuations enter at a point x depends on the density of the respective other particles in a neighborhood (sensing region) of the point x . Specifically, we use the so-called sensing radius, $r_{s}$ , so that in the limit N→∞ the spatially averaged densities, ${\tilde{p}}_{A,B}\left(x\right)$ in Equation 1 are (Schimansky-Geier et al., 2021)

$${\tilde{p}}_{A,B}\left(x\right)=\frac{1}{2r_{s}}\int_{-r_{s}}^{r_{s}}dx^{\prime }p_{A,B}\left(x+x^{\prime }\right).$$ (2)

Averaging close to the boundaries is implemented in a reduced window over an inside range of the respective densities; see below for an explanation. The case of local interaction where ${\tilde{p}}_{A,B}\left(x\right)=p_{A,B}\left(x\right)$ corresponds to $r_{s}=0$ .

Our model belongs to the broad class of active matter systems, ensembles of particles that continuously consume energy and therefore operate out of equilibrium (Romanczuk et al., 2012; Marchetti et al., 2013). In Equation 1, noise represents fluctuations in the internal drive of the particles. The time-dependent local density of the opposite species modulates each particle’s effective diffusivity (noise intensity). This reciprocal motility control represents energy influx at the microscopic scale, breaking detailed balance. Consequently, the observed steady states are maintained by a sustained flux of energy.

For the nonlinear function, f(p) , that determines the dependence of the noise intensity on the density of the other particle species, we choose a logistic function (Heinen et al., 2024),

$$f\left(p\right)=\frac{1}{1+\exp\left[-\alpha \left(p-p_{0}\right)\right]},$$ (3)

a function that monotonically grows with its argument (hence, the fluctuations driving the A particles at x become stronger when there are a lot of B particles) but shows a saturation: the noise intensity cannot grow unbounded, as was the case for the quadratic function used in Schimansky-Geier et al. (2021). We set the maximum diffusion coefficient (or noise intensity) to one, noticing that a value different from one can be absorbed by simply rescaling time. The positive parameters α and $p_{0}$ in Equation 3 determine the slope and the inflection point of the sigmoid, respectively.

Implementing the average of the densities as estimated from simulations of a finite number of particles deserves some comments, see Schimansky-Geier et al. (2021) for details. In our numerical simulations, the sensing radius accounts for a moving average of binned probability density functions (PDF). For each bin and a given time instant, the PDF is estimated by the number of particles that have fallen into this bin. To estimate ${\tilde{p}}_{A,B}$ , these PDF values are averaged over s bins to the left and s bins to the right, i.e., over 2s+1 bins, except for bins close to the boundaries. With the bin size Δx=2/(m−1) , this implies a sensing radius of $r_{s}=s\cdot \Delta x$ in Equation 2. The averaging window size is shrunk near the endpoints to include only existing bins.

In the limit of infinite particle numbers, N→∞ , the dynamics of the system is described by coupled nonlinear and nonlocal Smoluchowski equations, which describe the evolution of the probability densities (Schimansky-Geier et al., 2021)

$$\begin{array}{cc} \partial_{t}p_{A}\left(x,t\right) & =\partial_{x}^{2}\left[f\left({\tilde{p}}_{B}\left(x,t\right)\right)p_{A}\left(x,t\right)\right],\\ \partial_{t}p_{B}\left(x,t\right) & =\partial_{x}^{2}\left[f\left({\tilde{p}}_{A}\left(x,t\right)\right)p_{B}\left(x,t\right)\right]. \end{array}$$ (4)

We emphasize that these *nonlinear* diffusion equations may permit more than one steady-state solution. We implement a numerical solution of these equations by a finite-difference scheme described in detail in Appendix B of Schimansky-Geier et al. (2021), testing different initial conditions to determine the number and stability of asymptotic solutions. The integration of the Smoluchowski equations was terminated once the probability distributions at two consecutive time steps differed by less than $10^{-8}$ at every spatial point x . The stationary value of the order parameter (time-independent) was then computed from the stationary probability densities.

As in Schimansky-Geier et al. (2021), the system admits a homogeneous steady-state solution $p_{A,h}\left(x\right)=p_{B,h}\left(x\right)\equiv 1/2$ (the interval length is 2). However, this homogeneous state may be unstable. To capture the emergence of inhomogeneity, we define the time-dependent order parameter

$$I_{h}\left(t\right)=\int_{-1}^{1}{\left[p_{A}\left(x,t\right)-\frac{1}{2}\right]}^{2}dx,$$ (5)

which becomes significantly larger than zero whenever the system is demixed. This quantity can be computed from both finite-population simulations and from numerical solutions of the coupled Smoluchowski equations.

As an additional measure of order in the system, we consider the instantaneous mean position of the A particles:

$$\left\langle x_{A}\right\rangle =\frac{1}{N}\overset{N}{\underset{i=1}{\sum }}x_{A,i}\left(t\right),$$ (6)

which should remain close to zero when the A-particle distribution is homogeneous, but will deviate if A particles preferentially occupy one side of the interval (negative for the left side, positive for the right). Compared to $I_{h}$ , an advantage of this measure is that it explicitly distinguishes between the two possible demixed states. Finally, we note that these order parameters are symmetric with respect to an exchange of A and B particles, i.e., they remain invariant (with respect to their absolute value) under the substitutions $p_{A}\to p_{B}$ in Equation 5 and $x_{A}\to x_{B}$ in Equation 6.

For finite N , we employed the Euler–Maruyama scheme in the Itô interpretation to simulate the stochastic differential Equation 1 numerically. With the sensing radius, the dependence on the integration of the time step, Δt , is weak: the larger $r_{s}$ (or s ), the weaker the dependence on Δt (Schimansky-Geier et al., 2021). For s=10 used in the following, the results for $\Delta t=10^{-3}$ and $10^{-4}$ can hardly be distinguished. In contrast, omitting the averaging via sensing radius altogether and using directly the PDF itself in Equation 1, leads to an exceptionally strong dependence on the time step, such that extreme simulation times are required, especially to measure the jump statistics between metastable demixed and mixed states. In the following, we use the parameters as given in Table 1 if not explicitly stated otherwise.

**TABLE 1:** Simulation parameters.

Parameter	Meaning	Value
α	Steepness of nonlinearity	8
m	Partition for PDF estimates	100
s	Sensing radius parameter	10
Δt	Time step	$10^{-3}$

The simulation time window was chosen to ensure a sufficient number of transitions between metastable states in the bistable parameter regime for reliable estimation of the mean residence times, the probability density, and the correlation function of the order parameter. For exponentially distributed residence times, the standard error of the sample mean $\hat{\tau }$ of M switching events is $\text{SE}\left(\hat{\tau }\right)=\tau /\sqrt{M}$ , where τ is the true mean residence time. By the central limit theorem, the relative margin of error of $\hat{\tau }$ at confidence level 1−α is $\epsilon =z_{1-\alpha /2}/\sqrt{M}$ , with $z_{1-\alpha /2}=\Phi^{-1}\left(1-\alpha /2\right)$ the standard normal quantile, see e.g., Bendat and Piersol (2011). Thus, the required number of switching events is $M\geq {\left(z_{1-\alpha /2}/\epsilon \right)}^{2}$ . Accordingly, the expected simulation window is $T_{\text{max}}\geq \tau M$ . For ϵ=0.1 and 1−α=0.95 this gives M≥384 . We used $M=10^{3}$ and $\tau =10^{4}$ , which results in $T_{\text{max}}=\tau M=10^{7}$ , the value adopted in our simulations, accommodating for possible deviations from exponential statistics and occasional long residence times. To allow the system to approach a steady state, we first integrated for a relaxation period of $T_{\text{relax}}=10^{4}$ . The system was then integrated for an additional $T_{\text{max}}=10^{7}$ , and the time-dependent order parameter Equation 5 was approximated using the trapezoidal rule to compute the integral.

## 3 State diagram for N→∞

We start with the numerical analysis of Equation 4 and find the asymptotic state(s) of the system by solving the nonlinear equations for various initial conditions on a grid.

**FIGURE 1:**
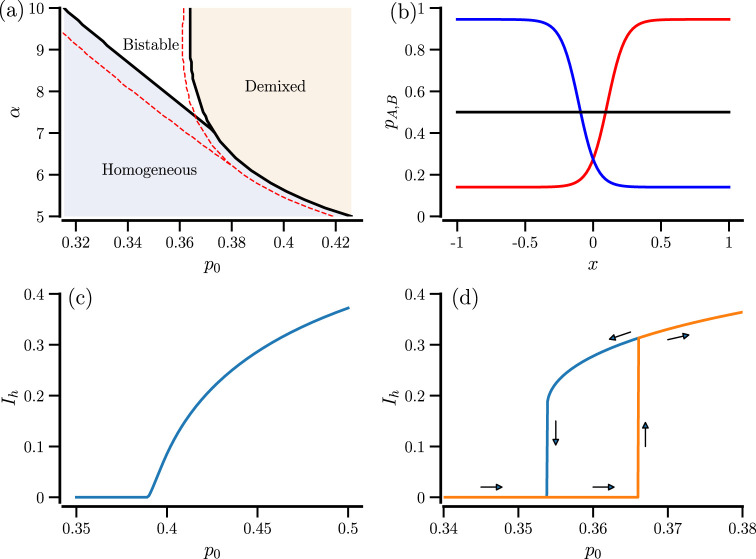
State transitions in the case N→∞ . **(a)** State diagram with bifurcation lines separating regions with a mixed (homogeneous) state, a demixed state, and with the coexistence of both mixed and demixed states (bistability). Non-locale case (black lines) compared to local case ( $r_{s}=0$ , red lines, for calculation, see Supplementary Appendix). **(b)** Steady states in the bistability region ( α=8 , $p_{0}=0.36$ ). **(c)** Continuous (second-order-like) transition for α=6 . **(d)** First-order-like transition for α=8 with forward (orange) and backward (blue) parameter continuation.

When we vary the parameters α and $p_{0}$ of the sigmoidal nonlinearity, we encounter parameter regimes that have been seen before with a quadratic nonlinearity in (Schimansky-Geier et al., 2021). Specifically, when the inflection point $p_{0}$ is low, and the function is not particularly steep (small α ), the homogeneous state in which both densities are well-mixed is the only stable state. When the inflection point is enlarged (but α is still small), we observe that the system demixes. There are regions where the A particles are in excess and the B particles are diminished in numbers, and there are other regions where it is the other way around; this is the demixed state. Now, beyond these two known regimes in which either a stable homogeneous or a stable demixed state exists, there is at large α and small $p_{0}$ also a regime of coexistence of both states. The lines of bifurcations are shown in Figure 1a; we also illustrate that the bistability does not hinge on a finite sensing radius (the red lines show the bifurcation for the case $r_{s}=0$ ). In Figure 1b, the new coexistence of demixed and homogeneous states is demonstrated for a specific parameter set: A and B particles might here be distributed in a homogeneous manner (black line) or by an increased density of A particles on the left and B particles on the right (because of the symmetry of the problem, there is the symmetric demixed solution with an excess of B particles on the left, etc.). We note that the densities, because of the finite averaging, do not add up to a constant, but there is an overall reduction of particles around x=0 .

As a consequence of the different parameter regimes, we see different types of transitions when the inflection point $p_{0}$ is increased, depending on whether the logistic function is shallow (α≲7) or steep (α≳7) . For a shallow logistic function, the transition shown in Figure 1c is continuous: close to the bifurcation, the order parameter $I_{h}$ is still close to zero, and demixing starts as a slight difference in the densities from the homogeneous state. For a steeper nonlinearity as illustrated in Figure 1d, we see a first-order-like transition with a homogeneous state $\left(I_{h}=0\right)$ that remains stable when $p_{0}$ is increased (orange line), then jumps abruptly to a pronouncedly demixed state, and continues further from there. Upon reversal of the parameter variation (blue line), we see a hysteresis effect. Namely, the system stays in the bistable state where previously the abrupt jump occurred before it jumps to $I_{h}=0$ at a smaller value of $p_{0}$ than the one at which the jump in the forward direction was observed.

The observed bistability in the system with an infinite number of A and B particles (corresponding to the Smoluchowski equations) raises the interesting question of what will happen in a system with a finite number of particles that is prone to fluctuations. We explore the bifurcation in a system with many but finite particles next and then turn to smaller systems in which finite-size fluctuations may trigger transitions between bistable states.

## 4 First-order-like transition in finite but large systems

**FIGURE 2:**
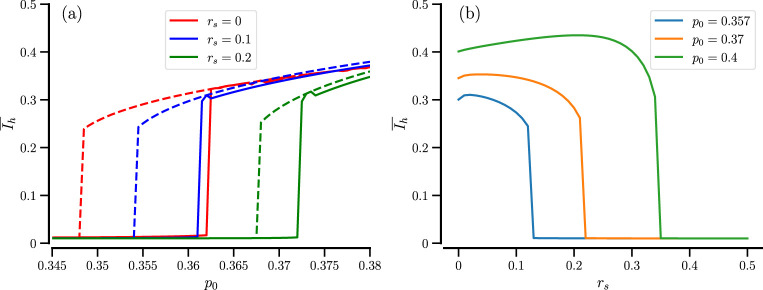
Effect of the sensing radius. **(a)** Time-averaged order parameter vs. $p_{0}$ for the indicated number of sensing radius, $r_{s}$ . Solid lines refer to a mixed initial state; dashed lines refer to a demixed initial state. **(b)** Time-averaged order parameter vs. $r_{s}$ for the indicated values of $p_{0}$ . The initial distribution of particles was in the demixed state. $N=10^{4}$ for both panels.

We now turn to a large system with $N=10^{4}$ of each A and B particle and integrate the stochastic differential equations Equation 1 with an α sufficiently large to encounter the above-discussed bistability. We use parameter continuation with different initial conditions: (i) the mixed state, in which $x_{A}$ and $x_{B}$ are uniformly distributed in (−1,1) , and (ii) the demixed state, where $x_{A}$ was distributed solely in (−1,0) , and $x_{B}$ in (0,1). From the two distributions of particles, we obtain time series $I_{h}\left(t\right)$ from which a time average $\bar{I_{h}}$ as well as its probability density, $P\left(I_{h}\right)$ , can be estimated. We illustrate the mean value in the case of a parameter continuation $p_{0}$ for the two initial conditions (i) and (ii) in Figure 2a by solid and dashed lines and for the values of the sensing radius $r_{s}$ (red, blue, green). It becomes apparent that the exact transition point $p_{0}^{*}$ as well as the width of the hysteresis depend on $r_{s}$ . With growing sensing radius, the critical inflection points move up, and the size of the hysteresis range shrinks. This is further explored in Figure 2b, where we show the time-averaged order parameter as a function of sensing radius for different values of $p_{0}$ . Also, here we observe an abrupt jump of $\bar{I_{h}}$ with increasing $r_{s}$ ; it is quite plausible that too much averaging (a too large value of $r_{s}$ ) will destroy the demixing mechanism. Interestingly, the order parameter for moderate increases may also cause a modest growth in the order parameter (cf., for instance, the maximum of the green curve in Figure 2b).

**FIGURE 3:**
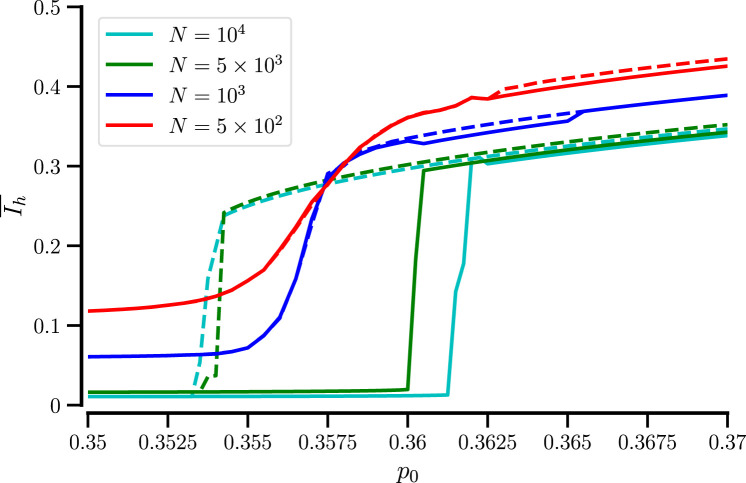
Hysteresis in the time-averaged order parameter is only seen for sufficiently large systems. Time-averaged order parameter vs. $p_{0}$ for the indicated number of particles, N . Solid lines refer to a mixed initial state; dashed lines refer to a demixed initial state.

How does the bifurcation plot depend on the number of used particles? This is exhibited in Figure 3 for our standard value of sensing radius, $r_{s}=0.1$ , and four different system sizes. The figure clearly shows hysteresis for large N ( $10^{4}$ and $5\times 10^{3}$ ), while for smaller N , the averaged order parameter displays a smoother dependence on $p_{0}$ . The latter dependence is expected if the system can jump between the bistable states within the averaging time window. We next explore the behavior of small particle numbers in more detail.

## 5 Switching between mixed and demixed state in the bistable regime for small N

**FIGURE 4:**
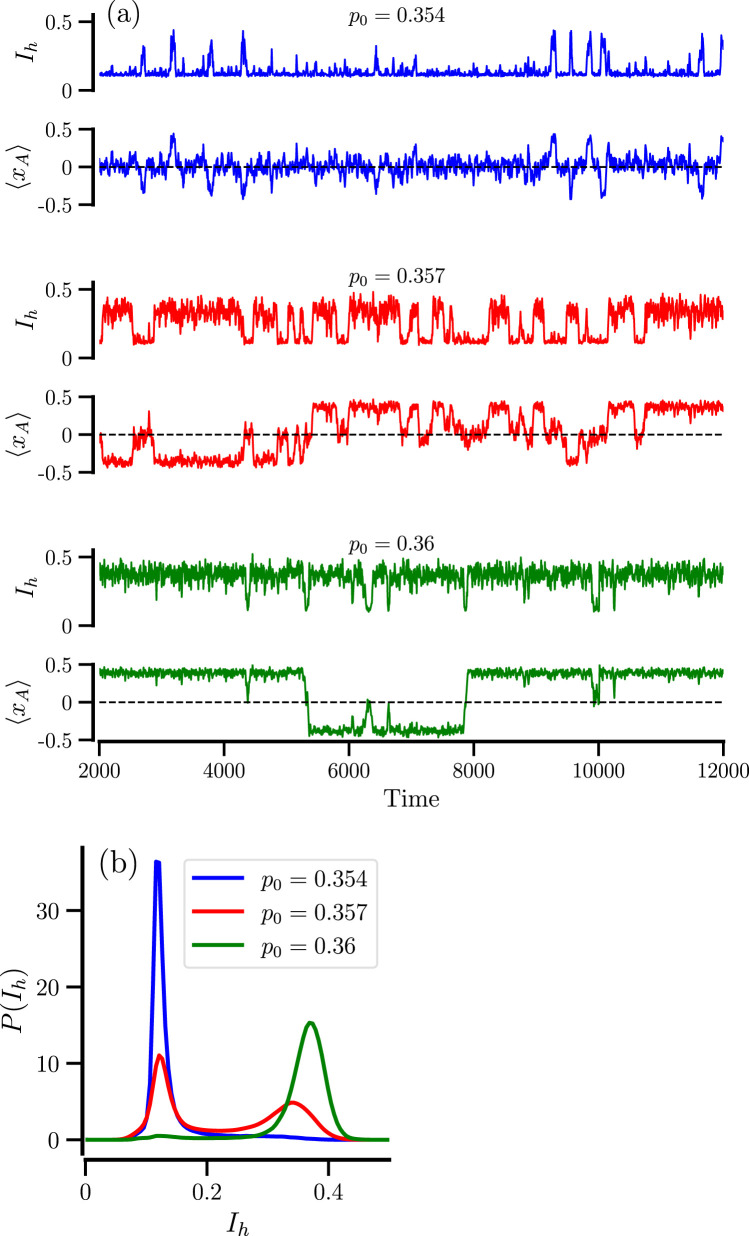
Stochastic transitions between mixed and remixed states for small particle numbers. Fragments of time traces of $I_{h}$ and $\left\langle x_{A}\left(t\right)\right\rangle$ **(a)**, and corresponding probability distributions $P\left(I_{h}\right)$ for full traces **(b)**, for $p_{0}=0.354$ (blue), $p_{0}=0.357$ (red), and $p_{0}=0.36$ (green) for N=500 particles. Time in the panel **(a)** and in the following is in arbitrary units.

For small N , e.g., for N=500 , the system exhibits switching between mixed and demixed states for parameter values within the range of bistability. Figure 4 exemplifies time traces of $I_{h}$ and $\left\langle x_{A}\right\rangle$ (panel a) and corresponding probability distributions $P\left(I_{h}\right)$ (b) for three parameter values. For $p_{0}=0.357$ (red line), the switching results in a bimodal distribution $P\left(I_{h}\right)$ .

From Figure 4a we can also conclude that both $I_{h}$ and $\left\langle x_{A}\right\rangle$ are viable measures of demixing. Furthermore, transitions between a demixed state with $\left\langle x_{A}\right\rangle <0$ (A particles preferentially on the left) and a demixed state with $\left\langle x_{A}\right\rangle >0$ (A particles preferentially on the right) or the other way around seem to have to pass through the demixed state first $\left(\left\langle x_{A}\right\rangle \approx 0\right)$ ; this is definitely the case for smaller values of $p_{0}$ but becomes less well visible for $p_{0}=0.36$ for which the mixed state is least stable.

**FIGURE 5:**
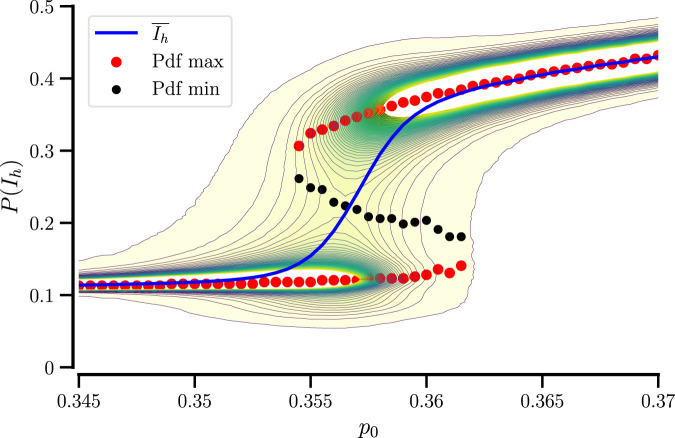
Probability distribution of $I_{h}$ vs. $p_{0}$ for N=500 . Red and black circles show positions of the maxima and the minimum of $P\left(I_{h}\right)$ , respectively; the blue line shows the mean, ${\bar{I}}_{h}$ .

Figure 5 shows a complete picture of $P\left(I_{h}\right)$ vs. $p_{0}$ , demonstrating a region of switching dynamics. Superimposed are the locations of maxima and minima of $P\left(I_{h}\right)$ (illustrating the bimodality in the intermediate region) and the smoothly varying time-averaged order parameter, ${\bar{I}}_{h}$ .

**FIGURE 6:**
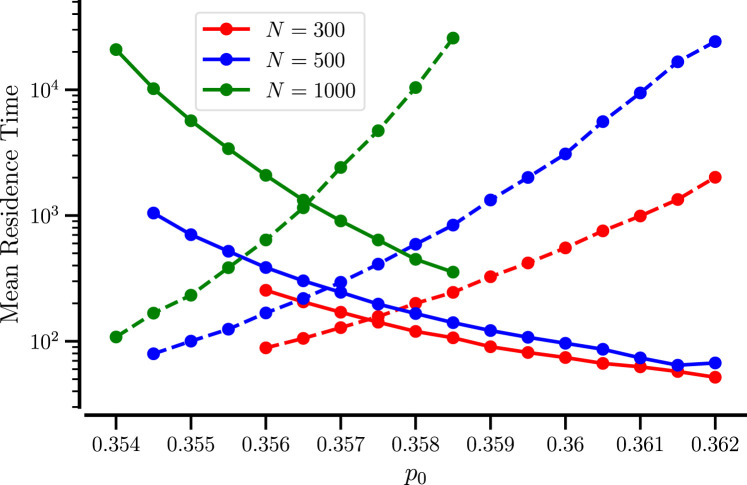
Mean residence times of mixed and demixed states for small systems. The mean duration of mixed (solid line) and demixed epochs (dashed line) vs. $p_{0}$ for the indicated values of N .

The stochastic transitions can be most appropriately characterized by the mean residence times in the mixed and demixed states. To this end, we extract many $\left(\geq 10^{3}\right)$ realizations of residence times from the time course $I_{h}\left(t\right)$ . The residence times were estimated as time intervals between a threshold crossing events lasting for at least $T_{\text{min}}=5$ , to avoid fast threshold recrossing. The threshold was determined as the local minimum of the probability distribution of the order parameter (black circles in Figure 5). Figure Figure 6 shows the averages over the two types of intervals for N=300 , 500, and 1000. The chosen range of $p_{0}$ values (with α=8 ) falls entirely into the bistable regime shown in Figure 1a.

Clearly, a larger number of particles implies ‘more waiting’: for N=1000 , the mean residence times are exponentially larger than for the smaller particle numbers. Furthermore, the mean residence time in the mixed (or homogeneous) state decreases upon increasing $p_{0}$ - this is quite plausible because we move the system away from the bifurcation boundary where this homogeneous state becomes the only stable state in the system and hence go the other way, we destabilize this state in the sense that we increase the system’s chance to leave this state. In contrast, the mean residence time of the demixed state increases - we stabilize this state by moving towards the bifurcation boundary at which the demixed state turns into the only stable state of the system.

There is a particular value of $p_{0}$ (which depends on N ) at which both mean residence times are equal. Here, both mixed and demixed states are roughly equally likely, and the histogram of $I_{h}$ may exhibit a pronounced bimodality. The particle number not only controls the finite-size noise in the system (in the sense of enlarging the mean residence times because fewer fluctuations can induce a transition) but also shifts the residence-time curves with respect to $p_{0}$ .

The slow back and forth between the different states of the system (especially at larger particle numbers and amid the bistable regime) may lead to long temporal correlations. We inspect their dependence on the system parameter by calculating the normalized correlation function of the order parameter

$$R\left(\tau \right)=\frac{\left\langle I_{h}\left(t\right)I_{h}\left(t+\tau \right)\right\rangle -{\left\langle I_{h}\left(t\right)\right\rangle }^{2}}{\left\langle I_{h}{\left(t\right)}^{2}\right\rangle -{\left\langle I_{h}\left(t\right)\right\rangle }^{2}}$$

and extracting its typical decay time, the correlation time by the following estimate

$$t_{\text{cor}}=\int_{0}^{T}d\tau |R\left(\tau \right)|.$$ (7)

**FIGURE 7:**
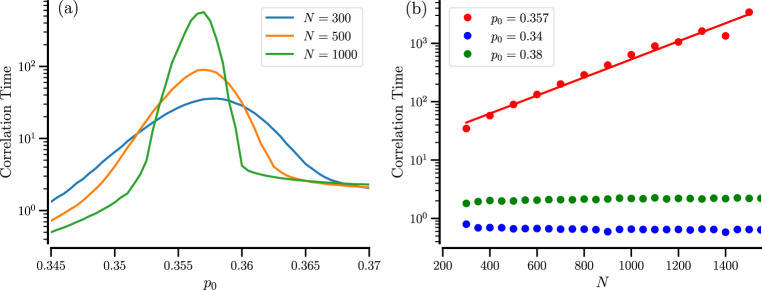
Correlation time of the order parameter. **(a)** Correlation time vs. $p_{0}$ for the indicated values of N . **(b)** Correlation time vs. N for the indicated values of $p_{0}$ . The solid red line shows the least-square exponential fit, $t_{\text{cor}}=\alpha e^{\beta N}$ , α=15.1 , $\beta =3.653\cdot 10^{-3}$ .

We note that the correlation time is easier to calculate than the residence times, as one does not need to define and use a threshold to determine the residence epochs. In Figure 7a, the correlation time Equation 7 displays a pronounced maximum at the sweet spot $p_{0}^{*}$ , corresponding roughly to the equal residence of the mixed and demixed states. Making the system more asymmetric, i.e., biasing it towards the mixed or demixed state, will reduce the correlation time. Similar effects have been observed for the diffusion coefficient of particles with a bimodal velocity distribution (that is actually proportional to the correlation time of the velocity process) (Lindner and Nicola, 2008; Lindner and Sokolov, 2016) and for the count variability in bursting neurons (Kullmann et al., 2022). In many of these systems, it was observed that the system size controls the height of the maximum, and the maximal correlation time grows exponentially with N . This is also true for our system, cf. Figure 7b.

Furthermore, when the number of particles N is increased, the maximum becomes more peaked such that outside a critical range of asymmetry, the correlation time decreases with increasing N (or is almost constant as is the case for the two extreme examples shown in Figure 7b in green and blue) while inside this range $t_{\text{corr}}$ increases strongly with increasing N . The range itself has a moderate dependence on N , as has also been observed for some of the other systems mentioned (Lindner and Sokolov, 2016; Kullmann et al., 2022).

## 6 Summary and discussion

We have inspected a minimalistic model of two populations of Brownian particles that mutually enhance by their presence the diffusion coefficient of the respective other population. Building on the results from Schimansky-Geier et al. (2021), as a new model ingredients we used here a sigmoidal nonlinearity characterized by an inflection point $p_{0}$ and a slope parameter α , replacing a simple quadratic nonlinearity previously considered in Schimansky-Geier et al. (2021). We showed that, besides the already known regimes in which either only a homogeneous (well-mixed) state is stable or a demixed state is stable, this model exhibits a novel regime in which these two states (mixed and demixed) can coexist. This was first demonstrated using the nonlinear Smoluchowski equations corresponding to a system with infinite particles.

Exploring the case of finite particle numbers using Langevin equations revealed the emergence of repeated stochastic transitions between the homogeneous (disordered) mixed state and a demixed state in which one kind of particle is in excess in one region while the other one is in excess in another region. Hence, in a strong formulation, the system switches back and forth between order and chaos, which we quantified by an order parameter that measures the deviation from a uniform distribution. We note that another measure would be the entropy of the two distributions: we have verified that the entropy indeed displays stochastic transitions between two values corresponding to the more ordered, demixed state and the more disordered, homogeneously distributed state (not shown). Repeated back-and-forth transitions between order and disorder have been observed experimentally in several systems (Pufall et al., 2004; Paul et al., 2022); interestingly, in the context of our work, the simplicity of the model leads to such transitions between high- and low-entropy states.

We have furthermore explored the transitions using the residence times and the correlation time of the order parameter and, specifically, their dependence on the bifurcation parameters and the system size. Typical for a symmetric bistable setup, the correlation time is maximized and displays an exponential growth in the system size.

It would be fascinating to observe this type of transition between mixed and demixed states in experimental systems of active matter. The new feature of our nonlinear dependence of the diffusion coefficient on the probability density seems to be rather realistic: diffusion coefficients can certainly not grow unbounded with the number of opposite particles (at some point, the presence of other particles may even hinder and limit diffusion), hence a saturation of D(p) is quite plausible. Whether this would also be possible in more complicated models of mutual motility dependence and in the experiments presented in Curatolo et al. (2020), remains an exciting question for future research. In particular, the model can be extended by incorporating a quorum-sensing effect by introducing a self-dependent diffusivity, in which, in addition to cross-density dependence, the motility of a species is affected by its own density. Our preliminary results indicate that quorum-sensing self-dependent diffusivity can drive clustering, the so-called motility-induced phase separation of active Brownian particles (Cates and Tailleur, 2015). However, the bistable mixing/demixing reported here relies on reciprocal motility control between two species.

Our logistic mutual diffusion model captures self-organized demixing and noise-induced transitions between mixed and demixed states, behavior that resembles a thermodynamic first-order phase separation transition as in liquid-liquid phase separation (LLPS) (Banani et al., 2017; Zeigler et al., 2021). Further, cross-diffusion systems can be recast formally as Cahn–Hilliard–type equations (Berendsen et al., 2017) and therefore may share phenomenological features with LLPS models (Laghmach and Potoyan, 2020; Li and Hou, 2022). Conceptually, however, our model is an active media system and is not an LLPS model. In our model, the demixing is driven by reciprocal modulation of diffusion rather than by intermolecular interaction energies, as in LLPS.

The observed behavior in our model is a form of self-organization—more precisely, a transient self-organization that repeatedly emerges and decays. It may be useful to regard the spontaneous formation of high-concentration domains as a dynamical network whose nodes exchange particles and either adjust their size (approaching a single-interface metastable state) or disaggregate (transitioning to the disordered state). Methods from the theory of adaptive networks (Gross and Sayama, 2009; Berner et al., 2023) could be used to extend and analyze our framework in settings where state-dependent (context-dependent) interactions are expected. In our model, “state-dependent coupling” refers to the cross-diffusion gains depending on the locally averaged density of the partner species. Beyond microbial consortia, analogous activity-dependent couplings occur in several physiological contexts, for example, cancer–immune crosstalk in tissues (Hsieh et al., 2022; Marzban et al., 2024) and organ–organ interactions discussed in Network Physiology (Bashan et al., 2012; Qi et al., 2024; Lehnertz et al., 2020; Ivanov, 2021). We emphasize that these connections are qualitative, as quantitative mapping would require system-specific variables and timescales.

Our results may be viewed in the broader framework of synergetics, originally proposed by Hermann Haken, wherein emergent macroscopic patterns and states in nonlinear systems are cast via a small set of order parameters that “enslave” microscopic degrees of freedom (Haken, 1977; Haken 1983; Haken, 2012). In particular, our model’s demixing phenomenon, driven by reciprocal diffusivity feedback, exemplifies the hallmark of self-organization: above a threshold in the control parameter (here, the sigmoidal strength), a new collective state emerges and coexists with the homogeneous state, leading to noise-induced transitions between these metastable configurations.

## Data Availability

The original contributions presented in the study are included in the article/Supplementary Material, further inquiries can be directed to the corresponding author.
