# Supplementary material for: Metastability in the mixing/demixing of two species with reciprocally concentration-dependent diffusivity
Source: Front Netw Physiol. 2025 Nov 17;5:1612495. doi: 10.3389/fnetp.2025.1612495 (PMC12665743; doi:10.3389/fnetp.2025.1612495)
Supplement: Supplementary file 1 [file Supplementaryfile1.pdf]

Supplementary Appendix for Metastability in the mixing/demixing of two species

DERIVATION OF BIFURCATION LINES WITH VANISHING SENSING RADIUS

The bifurcation lines in Figure 1a of the main text can be determined from the solutions of a transcendental equation when a vanishing sensing radius r_s is considered. This is similar to the calculations performed in Schimansky-Geier et al. (2021) but outlined in the following for the sake of completeness.

For a vanishing sensing radius, the averaged density becomes identical to the density itself, $\lim_{r_s \to 0} \tilde{p}_{A,B}(x) = p_{A,B}(x)$, and the stationary Smoluchowski equations (taking into account the reflecting boundary conditions) to

$$f(p_B(x))p_A(x) = C_A,$$
$$f(p_A(x))p_B(x) = C_B,$$

where C_A, C_B are constants that we choose to be equal (corresponding to solutions that we encounter in simulations). We furthermore assume that the solution $p_A(x)$ attains at most two distinct solutions, p_L and p_H, that are connected by the normalization condition $p_L + p_H = 1$ and that if p_A is high, the density p_B is low, and the other way around. This leads to the equation

$$\frac{p}{f(p)} = \frac{1-p}{f(1-p)}$$

or, after inserting the logistic function and expressing the derivation of the elevated or reduced densities by a parameter ε, $p_H = 1/2 + \varepsilon, p_L = 1/2 - \varepsilon$, we find that the solutions of the above equation can be expressed by the zeros of the function

$$G(\varepsilon) = \left(\frac{1}{2} + \varepsilon\right)\left[1 + e^{-\alpha\left(\frac{1}{2} - p_0 + \varepsilon\right)}\right] - \left(\frac{1}{2} - \varepsilon\right)\left[1 + e^{-\alpha\left(\frac{1}{2} - p_0 - \varepsilon\right)}\right],$$

a function that is obviously odd, $G(\varepsilon) = -G(-\varepsilon)$ and should be considered in the interval $\varepsilon \in [-1/2, 1/2]$.

The function is shown for different parameters α and p_0 in Fig. S1, illustrating the occurrence of metastable solutions related to the zeros of $G(\varepsilon)$. The line $p_0(\alpha)$ at which the zero loses stability is determined by the vanishing slope of the function $G(\varepsilon)$ at $\varepsilon = 0$, leading to a simple equation the solution of which is

$$p_0 = \frac{1}{2} - \frac{1}{\alpha}\ln\left(\frac{\alpha}{2} - 1\right).$$

This describes the part of the red lines in Figure 1a of the main text that separates the mixed and demixed regimes (below $\alpha = 6$) and the bistable and demixed regimes (above $\alpha = 6$).

The remaining bifurcation line separating the bistable from the well-mixed regime is determined by the emergence of a novel zero of $G(\varepsilon)$ when the function touches the ε-axis. This leads to the two conditions

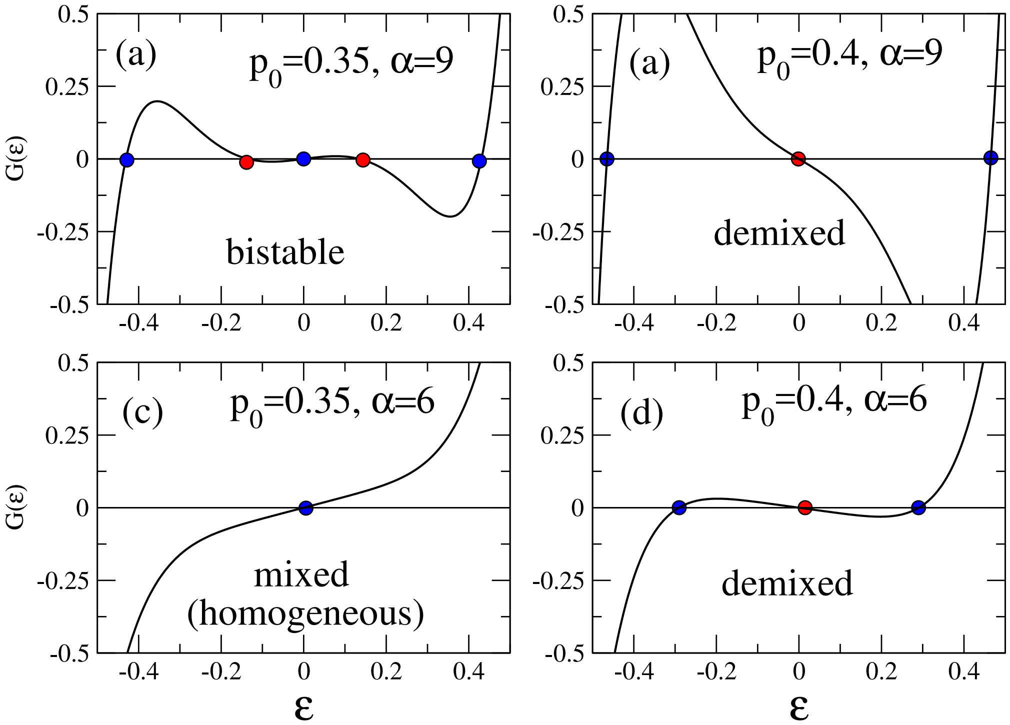

Figure S1. A function the zeros of which determine the possible solutions of the density for $r_s = 0$. The function $G(\varepsilon)$ (where ε quantifies the deviation from the uniform distribution) is plotted for different combinations of parameters as indicated in (a)-(d). The parameters are on the four corners of a rectangle in Figure 1a of the main text: the right ones ($p_0 = 0.4$) are both in the regime of demixed densities, whereas the left ones ($p_0 = 0.35$) are either in the well-mixed regime of homogeneous densities ($\alpha = 6$ in (c)) or in the bistable regime ($\alpha = 9$ in (a)), in which both the homogeneous distribution as well as the demixed solutions are stable. We indicate stable (unstable) solutions by blue (red) circles.

$G(\varepsilon; \alpha, p_0) = 0$ and $dG(\varepsilon; \alpha, p_0)/d\varepsilon = 0$, which we numerically solve at a given α for ε and p_0, yielding the second red line in Figure 1a of the main text.

The stability of the homogeneous state in the mixed and bistable regimes, and its instability in the demixed regime, can be proven as was done in (Schimansky-Geier et al., 2021).

REFERENCES

Schimansky-Geier, L., Lindner, B., Milster, S., and Neiman, A. B. (2021). Demixing of two species via reciprocally concentration-dependent diffusivity. *Phys. Rev. E* 103, 022113
